# Cases of Organic Diseases of the Womb and Its Appendages

**Published:** 1847-08

**Authors:** Walter Channing


*Cases of Organic Diseases of the Womb and its Appendages.* By WALTER CHANNING, M. D.—Chronic organic affections of the womb and of its appendages have long been the reproach of medicine. They, many of them, tend to death, are malignant, and, having a bad name, little more is done for them than to make them as tolerable to the sufferer as well may be. Dr. Wm. Hunter, in a manuscript lecture of his on cancer of the womb, in my possession, makes many sensible remarks on the best mode of retarding those processes which always end in death. Thus he advises abstinence from animal food, except fish now and then, and of these recommends such only as are of white flesh, and have the least flavor—are the least stimulating, in other words. He is particular in his rules about dress, and especially directs that women with functional or organic disease of the womb or its appendages, and those, too, who would escape these, should wear flannel drawers. Rest, also, is among his prescriptions, and sarsaparilla his principal medicine. But Dr. H. says nothing about cure. His means have in view only to retard the advance of destructive processes, as ulceration in cancer, and so to prolong life. He does not look to cure, as among the purposes of treatment in such diseases. He would mainly labour to make a life of daily suffering more tolerable than without his agency it might be.

Osiander, of Gottingen, labored to cure, to remove radically, malignant diseases of the womb. He cut away diseased portions, and with a bravery which was always tempered with wisdom, and guided by the best knowledge of what he meant to do, and how it should be best done. He was often successful. McDowell, a physician in our western wilds, determined to do something to prevent the fatal issue of diseased ovaries, and in pursuing this purpose extirpated them, with a success, and in numbers, and under circumstances, which made an epoch in that department of surgery. Dupuytren and others followed Osiander in excising the mouth, the neck, or larger portions of the womb, but they were not very successful. Lizars, and quite lately Gray, have followed McDowell in his operations, but with less success.

Occasionally a case is referred to, of removal, either spontaneously, or by, or after the employment of medicine, of these diseases. I have thus known a cauliflower excrescence removed by ligature, without return of the disease. I have known very striking effects follow the internal and external use of medicine for tumors which for the most part are regarded as incurable, and which are passed by without exciting any remedial regard. Sir Astley Cooper somewhere speaks of the professional negligence of these diseases. Is it right to abandon their subjects, simply because such diseases have got a bad name? I have always treated them as I would other diseases. My attendance on them has been regular, and means have been put into use. The attendance has ceased, because at length it has appeared obvious that no benefit was coming of treatment, or the patient, or more frequently her friends, have thought farther care, or farther expense, unnecessary.

I will, in more or less detail, refer to some cases of reputed malignant diseases of the womb, and of its appendages, which have come under my notice, and for which means have been used, with a view to curative agencies.

*Cases of Cauliflower Excrescence.*

Case. I.—Mrs. ———. Tumor small, insensible, springing from the os uteri. Drain of *watery fluid* from vagina constant and great; occasionally hemorrhage; much exhaustion, and evident sinking from disease. A ligature was applied round the tumor. It came away, bringing a few shreds only of what it had surrounded. Discharge ceased. Upon examination no tumor was to be discovered. The os uteri from whence it sprang, was found healthy. Strength returned, and the patient after a time left the city without complaint. I have not heard of her since.

Case II.—Mrs. M———. The history of this case is very much the same with that above given. There was an insensible tumor springing from the os uteri, irregular in its outline, or rough from small projections. *Watery drain* constant from vagina—at times hemorrhage. Ligature was applied, and in a few days came away, bringing scarcely anything with it. The noose had sticking to it some very small remnants of the large tumor which it had surrounded. This woman recovered perfectly.

Case III.—Mrs. ———. This woman had a tumor like those above described, and with like symptoms. She died exhausted by discharge of water and hemorrhage. I saw her after death. Scarcely any appearance of tumor was discovered. In this case ligature was not applied.

Case IV.—Mrs. K———. I was desired in December, 1846, to visit Mrs. K. I found her in bed, thin, pale and sallow, and apparently suffering from some chronic and exhausting disease. She said she was over 40 years old, had been married ten years, and had never had children. Her complaint, she continued, was a tumor of the womb, which was of more than a year's notice by her. At times much hemorrhage occurred from it, and at all times a *watery* discharge. Of late this had become so great as to exhaust her exceedingly, and she thought that it would be impossible for her to survive many weeks if it continued. It was sufficient to wet forty large towels a week, sometimes eight in a day. It was water, without colour, without smell, and without mucus. The disease was in no sense, or in itself, a painful one. It pressed against the sacrum and produced pain in that bone, and it obstructed much the rectum, and so troubled its functions. She added, that within a short time emaciation had rapidly increased, and her strength had failed. She wanted to live. She would endure anything that she might live. I have heard the desire to live expressed by many, and when death was near at hand by cause of sudden diseases, or accidental occurrences, without precursory disease; but I never heard it utter itself with such emphasis as it spoke from that woman. She had a face which expressed in the strongest manner her whole feelings, and having passed her hand over it as if to remove from it whatever might diminish the power of what she had to say, bent her eyes upon me as I leaned over the foot-board of her bedstead, and said, "Have you the heart, Sir, to do an operation which may save life, though it be at the risk of taking it? That is the question which I want answered, and in the most distinct manner." I said that I believed I was equal to my whole professional obligation, and would state to her what might be my duty specially to her, after I had learned by an examination what was her disease, and that I would act as that duty seemed to me to demand. She said that her case had already been examined, but no operation recommended, but she could not yet give up. She begged me at once to make the examination which was to settle the course I was to pursue.

Upon examination I found a tumor filling the vagina, and which was most developed in its sacral aspect. It was hard and perfectly insensible. It was not regular in its outline, but had projections, and superficial, and deep sulci between its rounded and ridge-like inequalities. It was of a greyish colour, and the discharge from it was an inodorous, colourless water. I said I saw nothing in the character of the tumor to forbid an operation. I described to her the operation, the ligature, and stated that it would give her little or no pain. I learned that a professional friend of great surgical knowledge and reputation had been asked to see her, but had not called, and said that I would request him to see her, as I wished to have his opinion of the operation I had in view. He called the next day, and wrote me that he thought the tumor presented no objections to the ligature, that an operation in the case might be done, but that inflammation might follow, and the tumor might re-appear. This opinion was stated to Mrs. K. She determined to submit to the operation, and to abide the issue.

A ligature was passed round the tumor, in the close of December, and it was purposely applied as high as it could be done. It was not found so easy to do this as was supposed, and as those who never made the attempt may imagine. The tumor filled the vagina. It was exceedingly irregular in its outline. The double canulæ of Gooch was used, and easily carried along the fingers, to the root of the tumor. The attempt now to separate the two, by keeping one fixed, and sweeping the other horizontally round the tumor, was embarrassed by many obstacles. It was at length done, and the silver clasps carried along the tubes to make them again one instrument. The ligature was now drawn tight and secured. Some pain followed this part of the operation, but soon subsided. It resembled the dragging sensation which I have heard complained of in other cases. The next day I found the instrument very far out of the vagina, and upon examination discovered that the ligature did not embrace the whole of the tumor, but had been carried by the canula into one of the deep depressions of the tumor, instead of encircling the base. I state this thus particularly, as a caution in applying the ligature round uterine tumors, especially malignant ones, which are much more apt to be irregular in their outline than are the non-malignant. Said a physician who very kindly assisted me in this operation, let those who are astonished at a failure in doing such an operation try to do it themselves, and they may learn that there are difficulties in the way they hardly dreamed of. The ligature was now removed, and then applied again, and with entire success. It was tightened every two or three days, and each time showed that there was a gain on the tumor. Some days the ligature could be drawn an inch before it stopped. Generally not so much. There was always pain on drawing the ligature. It, however, was never a permanent pain, or of any long continuance. An opiate was often given to remove it, and doubtless made the time of suffering shorter. The instrument began at length to project farther and farther from the vagina. The ligature showed by its length that very little of the tumor remained encircled by it. I now examined the vagina, and found it empty. I could feel nothing but the canula, and a small loop of ligature encircling a portion of the original tumor which had not disappeared. The os uteri, excepting this spot which looked to the sacrum, was perfectly smooth and even in its outline, not a shred of tumor remaining except at the point referred to, and this was firmly within the grasp of the ligature. There was no discharge from the vagina. There had been scarcely any since the ligature was applied. What discharge did take place was the liquified tumor as it was cast off by the living textures from which it had been separated by living agencies. Nothing like a tumor had been thrown off. The ligature was now daily tightened, and I daily looked for its coming away. On the twenty-second day from the use of the canula, I found Mrs. K. in unusual spirits, and hiding something under the bed-clothes as I entered. I asked what it was. She at once drew out the instrument, which she said had come away that morning, and which she was now rubbing very carefully to restore to it its original brightness. I asked if anything had come away with the ligature. She replied, yes, and showed me a thin small mass, like dense membrane, on a towel. Upon examination I found that portion of the tumor remaining, round which I felt the ligature, of the size say, of one joint of the thumb, projecting from the posterior lip of the os uteri. I asked how it happened that the instrument had come off before separating the tumor. She said that she felt a strong desire to rise from bed to evacuate the bowels, and that, in suddenly moving, the instrument had been caught in the bed-clothes and was torn away from the vagina, giving her exquisite pain. I made no attempt to re-apply the ligature, for such was the stiuation of the remaining portion of the original disease that I did not believe that a ligature would hold on it.

Mrs. K. now rose from bed. Took exercise. Got excellent appetite. Gained flesh, and felt well. Not the smallest drain occurred from the vagina of water, of pus, or of blood. Her feet and ankles swelled, but at length this swelling disappeared. But after some weeks signs showed that the disease had returned. There was again water, and blood, and pain. She began to fail, and in five months after the operation she died. To the last she never regretted the operation. It had lengthened life and enabled her to enjoy it. Cheerfully would she submit again to the same measure, if it promised her the least chance of continued existence. I could offer her no inducements to submit to it. Her power of living was well nigh gone, and she sunk and died.

*Cases of Uterine Polypi removed by Ligature.*

Case V.—Miss. S., aged 18. Came under my charge last summer. She was perfectly anæmic. I have seen many instances of this disease, but in no one do I remember its physiognomy to have been more strongly marked. The lips, gums, tongue, inside of cheeks, carunculæ lachrymales, nostrils, every texture was blanched. She had suffered from uterine hemorrhage for about four years. She had grown tall in that time, the time of growing, and was not emaciated. Other signs were palpitation on exertion or mental emotion, with dyspnœa, a sharp, quick, and frequent pulse, pink-colored veins, ringing or rather buzzing in the ears, and a throbbing of the cerebral arteries, compared to the sound of the discharging pipe of a steam engine. Stomach at times irritable. Great feeling of lassitude, and of exhaustion. The catamenial periods were attended by excessive flow, and in the intervals hemorrhage was frequent. She had been recently examined, and a tumor projecting out of the os uteri diagnosed. Upon examination I found the tumor to be very hard, insensible and small, of the size of a sma l pullet egg. Hemorrhage attended, and followed the examination. I could not reach the pedicle, if the tumor had one. I was disposed to consider it as springing from the cervix with a broad base. The ligature was applied. I tried different canulæ in this case, and though the tumor was not large, I found the largest instrument to answer best.

Pain was produced by tightening the ligature. As soon as pain occurred, the ligature was fastened where it was. Some soreness of the abdomen was complained of next day, and the instrument was not touched. The next day it was tightened, and pain again produced. Soreness of abdomen again occurred. I was satisfied that the tumor was insensible, but the neck being very short, if any existed, the strain of the whipcord was upon the womb, at the spot whence the polypus sprung. This explains the pain and the abdominal tenderness. In about a week the tumor and instrument came away. The polypus was perfectly white. I scarce recollect so white an animal texture. From this time hemorrhage ceased. The catamenia became regular, and the patient is now perfectly well. The tumor was accidentally lost.

Case VI.—Mrs. W., aged about forty ; has children. Has suffered greatly of late from uterine hemorrhage, and the exhaustion induced. Of late she has been obliged to keep still, and in bed, as in this way only can she prevent flooding. She suffers many of the symptoms given in Miss S.'s case, but not in so severe a degree. Within a few weeks her case has become alarming, and her physician, upon examination per vaginam, discovered a tumor projecting from the osuteri I was desired to see Mrs. W., and upon examination detected a tumor, in, but hardly projecting from, the os uteri. It was hard and insensible. It was closely embraced by the os uteri, so close that I could only pass a probe between them. It seemed very much as the glans penis looks in a severe case of phymosis. The woman was bleeding badly every day, but it was not possible to operate. It was advised to give her ergot in ten-grain doses, as often as five or six times daily, if her stomach would bear it. This was done, and pains in region of womb produced, and after a few days I found the tumor so much out of the womb as to allow the use of the ligature. It was applied. Hemorrhage at once ceased. Pain in the womb and abdomen was produced by tightening the cord, as in Miss S.'s case. But no untoward complication ensued, and in four days the tumor came away; since which, Mrs. W. has done perfectly well. The tumor was very firm and of a deep red color.

*Cases of Ovarian Tumor.*

Case VII.—Mrs. ———; married, has children. I saw this patient with Dr. Hanaford, A tumor, filling most of the lower part of the abdomen, presented itself. It was unequal in its outline, as if composed of many tumors. It was felt in the vagina, and so far filling it as to press the neck of the womb firmly against the symphysis, giving much trouble in passing urine. The uterine periodical function continued. Much suffering attended this disease. At times acute inflammatory attacks. The health had sunk, emaciation had occurred, the situation of the patient seemed hopeless. In its treatment constant efforts were made to sustain the patient while means were used to keep inflammatory processes in check, and to prevent increase of the tumor. Leeches, vesication, iodine ointment, hydriodate internally, sol. muriate of lime, &c., were among the means employed. At length a new symptom showed itself, which was the precursor of recovery. An involuntary and copious liquid discharge took place from the rectum. Some of it was collected in a vessel and examined. It was a perfectly transparent, dense, gelatinous liquid, very adhesive, and having a distinct albuminous odor. There was not the least trace of fecal smell in it at any time. The discharge went on. The tumor grew less. The pains which Mrs. ——— had so long suffered, ceased. She regained appetite, strength, flesh, and is now well. Is not this case of some interest in this regard, that it shows how a disease of the ovary, consisting of a fluid deposit, and threatening life, may disappear, and recovery happen, where opportunity is offered for a constant discharge of the fluid as it forms. The sac or sacs thus have an opportunity to contract. New processes occur in them from exposure to the air, and at length the sac disappears. What is done by a rude surgery, the pulling away, or cutting away the sac, or removal of the tumor by excision, is done by a gentler and wiser hand, and recovery follows. May not the ovary be punctured through the rectum, or vagina, and the natural surgery be thus in some sort imitated?

Case VIII.—E. L., about 30, unmarried. Has a large tumor oc cupying one side of the abdomen, extending from the right iliac region beyond the median line towards the left, and above the umbilicus. Tumor apparently solid. I say apparently, for it is not easy to diagnose the liquid contents of such tumors, if liquids be in them, especially if the walls be thick, and the cavity within be formed of two or more non-communicating sacs. Examination per vaginam discovered a solid tumor filling most of the pelvis, and which seemed to be a part of the general mass above described. This patient suffers much from her disease. At times the bladder and urethra are so compressed that urine cannot pass. At times the rectum is obstructed. The result is most severe pain and distress throughout the abdomen. The trouble here is spasmodic, and at times exactly resembles violent colic. In the intervals of these attacks she was able to do some work in a family, to walk the streets, &c. She had for some time been engaged to be married. A desire had been manifested to have the engagement broken off. I was consulted. I gave an opinion decidedly in favor of the measure; and had there been a legal question raised, I should have felt it my duty to state that such an "impediment" (I use the word in its ritual technicality) existed as made the state of marriage improper. I remembered well a case of recent marriage, in which the husband desired my advice on account of important disease, not known to exist before marriage. I discovered that a tumor absolutely filled the whole cavity of the pelvis, and which, if it continued to grow, would in no long time be seen externally.

I began an active course of treatment very soon after I saw this case of E. L. The diet was regulated, so was exercise and rest. Iodine ointment was constantly applied over the whole extent of tumor; and sol. mur. lime was steadily given. She took of this larger doses than has any other patient to whom I have prescribed it. The dose amounted at last to about three hundred drops, three times a-day. Leeches were used whenever pain existed in the tumor, and occasionally counter-irritation was employed. This course was persisted in for a long time. The tumor gradually diminished. This was ascertained by careful admeasurement. The strength returned. She gained flesh. She has for a long time passed from my professional care, but I occasionally see her, and always learn that she has no complaint. I have made no examination for a long time; but I feel sure that if the disease had become active again, I should have known it.

*Remarks.*—Mrs. K.'s case, No. IV., occupies a large space in this record. It was a case of much interest. It involved some important principles. Are we authorized to operate in such cases? How far are we to be influenced by our patient in deciding such a question? Here was entire faith in what was to be done. It was faith in it as the only means of life—offering the only chance, however faint, that was of good. It was associated with an intense desire to live. The disease, called by C. M. Clarke and others malignant, has within a few years been successfully treated by ligature. The immediate effect of ligature in this case was good. It lengthened life, and made life comfortable. I do not recollect a circumstance in this case which brings with it any regret that the operation was done. A question has been asked above, how far shall the physician be influenced in his judgment in treating disease by the demands, or wishes of his patient? As a rule, and the demand existing by itself, there being nothing in the case which promises good from any known agencies, he is not to be influenced at all by the wishes of his patient. But cases do occur which form, or are regarded as exceptions to the rule, and the physician is, and will be, governed by them. I remember, when a hospital pupil, a sailor entering the hospital for an affection of the heel which renderd him perfectly useless, and for years, under all sorts of treatment, had made him a great sufferer. He came to have his leg cut off. He had hobbled over the country, and applied for the *radical* treatment in vain. His leg, heel, foot, all, had not the slightest appearance of disease about them. His demand was heeded, his request granted, and he had the operation done with apparently as little suffering, certainly with as little complaint, as if he had been under the fullest power of ether.

I remember another case. It occurred in Scotland. An unmarried woman had a swelling of the abdomen of great size. It troubled her extremely. It did so principally in preventing her getting employment, she being supposed pregnant. This was a sore charge, and most grevious in its consequences, for she depended on work for her living. She roamed about to have an operation done on the abdomen, by means of which a large tumor, which surgeons regarded her trouble to be, might be removed. She applied in vain, till at length Mr. Lizars, then of Edinburgh, consented to do the operation. An incision was made of great extent, into the peritoneal cavity. *But no tumor was found.* The abdominal intumescence was owing entirely to a very large deposit of fat in the walls of the abdomen, and a very fat omentum.

In deciding the question of an operation in the cases referred to, the surgeon acts upon his own responsibleness, and his sense of this must determine for him what the practice must be.

A word more. In cases V. and VI., of polypi of the womb, it is said that pain was complained of when the ligature was tightened, and that soreness of the abdomen followed. The same was said of tightening the ligature in Mrs. K's. case. Now these three tumors are *insensible*, wholly so, certainly were they in the cases referred to. Whence, then, the pain? I have already answered this question, by supposing that it happened from the nearness of the ligature to the proper substance of the womb itself. The tumors had no pedicles. They sprung from broad bases, and the ligature was applied very near to the base. The womb, though no portion of it was included in the ligature, did receive pressure from the ligature applied so near to it. Now it is not important that the ligature should embrace polypi round the pedicle, or very near the base, especially in cylindrical or globular polypi which have no pedicle. If the ligature be applied at such a distance as not at all to affect the womb, the *whole of the*

*tumor will drop off*, just as does the whole of the umbilical cord, let us leave what amount we may adherent to the abdomen.

But if the pain on tightening the ligature be such as to attract regard—if it be accompanied by the constitutional or local symptoms of uterine or peritoneal inflammation, slacken or remove it at once, and treat the disease it has produced at once with appropriate remedies. When applied again, select a spot more distant from the womb. Make the pressure less severe if pain again accompany it. If there be no pain, make it firm enough to strangulate the tumor at once, or as far as may be, as less constitutional trouble is apt to arise from such an operation, than from a less perfect and positive one.—*Boston Med. and Surg. Journ.*

